# Online health information seeking behaviors and self-reported health among Chinese university students: associations and sex differences

**DOI:** 10.1590/1980-220X-REEUSP-2025-0253en

**Published:** 2026-02-23

**Authors:** Jie Chen, Hua Tian

**Affiliations:** 1 Xinyang Normal University, School of Marxism, Xinyang, China.; 2 Xinyang Normal University, College of Tea and Food Science, Xinyang, China

**Keywords:** Health Information Interoperability, Health Evaluation, Sex Characteristics, Students, Interoperabilidad de la Información en Salud, Evaluación en Salud, Características Sexuales, Estudiantes

## Abstract

**Objectives:**

Aim to explore the associations and sex differences between online health information seeking behaviours (OHISBs) and self-reported health among university students.

**Methods:**

Cross-sectional, analytical study carried out in the 5,408 university students from Xinyang Normal University, who responded questionnaire in Wenjunxing as valid participants. t-tests and binary logistic regression analysis were performed using SPSS.

**Results:**

Sex differences in OHISBs were found. Females most liked food nutrition and diet-related OHI (p < 0.001), while males most liked physical exercise OHI (p < 0.001). For specific OHISBs, finding information about hospitals or doctors (OR = 1.785, [CI95% = 1.212–2.628], p < 0.01), and online reservation of health care projects (OR = 2.491, [CI95% = 1.056–5.876], p < 0.05) had significant impacts on females’ self-reported health, and finding information about hospitals or doctors (OR = 2.171, [CI95% = 1.035–4.551], p < 0.05) had a significant impact on males’ self-reported health.

**Conclusion:**

Findings of sex-differentiated online health seeking and the benefits of proactive search indicate that effective digital interventions for students must combine gender-specific strategies with behavioral empowerment.

## RESUMO

**Objetivo:**

Explorar as associações e diferenças entre os sexos no que diz respeito aos comportamentos de busca de informações online sobre saúde (CBIOSs) e à saúde auto-relatada entre estudantes universitários.

**Métodos:**

Estudo transversal e analítico realizado com 5.408 estudantes universitários da Universidade Normal de Xinyang, que responderam ao questionário em Wenjunxing como participantes válidos. Foram realizados testes t e análises de regressão logística binária utilizando o SPSS.

**Resultados:**

Foram encontradas diferenças entre os sexos nos CBIOSs. As mulheres gostaram mais de IOS relacionados à nutrição alimentar e dieta (p < 0,001), enquanto os homens gostaram mais de IOS relacionados a exercícios físicos (p < 0,001). Para CBIOSs específicos, encontrar informações sobre hospitais ou médicos (OR = 1,785, [IC95% = 1,212–2,628], p < 0,01) e reservas online de projetos de cuidados de saúde (OR = 2,491, [IC95% = 1,056–5,876], p < 0,05) tiveram impactos significativos na saúde autoavaliada das mulheres, e encontrar informações sobre hospitais ou médicos (OR = 2,171, [IC95% = 1,035–4,551], p < 0,05) teve um impacto significativo na saúde autoavaliada dos homens.

**Conclusão:**

As descobertas sobre a busca por saúde online diferenciada por sexo e os benefícios da busca proativa indicam que intervenções digitais eficazes para estudantes devem combinar estratégias específicas de gênero com empoderamento comportamental.

## INTRODUCTION

Since the onset of the Coronavirus Disease 2019 (COVID-19) pandemic, more and more people have turned to the internet to search for health-related information^(1)^. The internet has become a valuable source of health information, offering resources on mental health^(2)^, general health^(3)^, physician-patient relationships, and health and wellness. Many health-related websites and apps provide consumer-oriented health information and additional functions^(4)^. Online health information (OHI) has become one of the most important sources of information^(5)^ for people of all ages seeking health information^(6)^. These include forums, blogs, online support groups, and recommendation links to information on all aspects of health^(7)^. Specific health information includes symptoms, diagnoses, treatments, and disease prevention, screening, and risk assessment. General information includes topics such as weight loss, healthy eating, and health tips^(8)^. For university students at Ondokuz Mayıs University who are not studying health sciences, the most frequently used online health information sources are forums and private hospital/clinic websites, while government websites are the least used^(9)^.

OHISBs are considered to have a positive impact on those seeking OHI^(10)^. According to a survey of two southern U.S. universities, 36.7% of students felt that online health information improved their health care significantly or somewhat. Significantly more female students (77.9%) than male students (68.6%) obtained health information online^(11)^. A total of 75% of health information seekers believed that the information had a slight or significant impact on their health treatment decisions, health maintenance methods, and health-related thinking^(12)^. For information on the novel coronavirus, male students were more likely than female students to believe that the internet alone could provide all relevant information^(13)^. In Nigeria, the presence of a chronic condition and gender were significantly associated with non-medical university students’ OHISBs^(14)^. A study showed significant associations and gender differences between Chinese university students’ OHISBs and their eHealth literacy^(15)^. However, studies have shown that university students lack the knowledge and skills to identify incorrect information and the obvious risks of their information-seeking behaviors^(4)^. Some online health information (OHI) may be inaccurate or misleading, causing unnecessary pain and anxiety to university students. Therefore, future curricula should include appropriate intervention measures, such as practical sessions, courses, and classes, as well as guidance on how to obtain reliable health information, especially for students in non-health-related fields.

Understanding gender differences in university students’ OHISBs is crucial for developing targeted interventions. Contemporary gender theory posits that gender is a diverse, dynamic, and culturally sensitive social construct, not merely a biological binary^(14)^. From this perspective, excluding non-binary identities fails to reflect real-world diversity and risks perpetuating misunderstanding and bias. However, this study was conducted in China, where the legal and social frameworks are binary and recognize only male and female biological genders at birth without a legally defined third gender. Therefore, this study’s scope is limited to a comparative analysis of males and females at birth and does not consider broader gender diversity^(15)^.

The following hypotheses are thus proposed:

H1: There are significant sex differences in OHISBs among Chinese university students that can inform the development and implementation of targeted intervention measures^(16)^.

Studies have found that patients with poor health tend to engage in OHISBs more frequently and have low electronic health literacy; thus, they may be more susceptible to unreliable OHI and the health risks it poses. Perceived unwellness could cause a higher level of OHISBs. Furthermore, higher levels of OHISBs are significantly related to lower self-reported health levels and higher levels of mental distress^(17)^. However, few studies have explored the relationship between specific OHISBs and the health of university students.

H2: Specific OHISBs have a significant impact on self-reported health and are associated with university students’ self-reported health.

In short, these findings can address the lack of research on sex differences in OHISBs and the relationship between self-reported health and specific OHISBs^(9)^ among Chinese university students. They can also provide a theoretical basis for future targeted intervention measures, especially for university students in non-health-related fields.

## METHOD

### Study Design

This is a non-experimental cross-sectional study.

### Population, Local and Setting

This study was conducted among university students majoring in the humanities, sciences, and engineering (unrelated to health sciences) at Xinyang Normal University in the city of Xinyang, Henan Province, China. From October to December 2022, university students were recruited to voluntarily and freely fill out a questionnaire. This study included OHISB questionnaires^(9)^, self-reported health questionnaires, and demographic information questionnaires.

### Sample Selecton Criteria

The study recruited 5,672 university students, from freshmen to seniors, to complete an anonymous online questionnaire. The questionnaire was created using Wenjunxing software. Then, the link to the questionnaire was sent to a group of WeChat teacher users, who were asked to share it with university students to recruit voluntary participants. The questionnaire is anonymous and voluntary, and participants can fill it out freely and independently on their computers or smartphones. There was no reward for participating in the study. Participants were native Mandarin speakers between 18 and 25 years old who were studying liberal arts, science, engineering, or arts. Those who did not complete the questionnaire were excluded (e.g., those who left all the questions unanswered, provided incomplete demographic information, or were not university students). In the end, 5,408 university students (a response rate of 95.35%) were selected as valid participants.

### Data Collection Instrument

Our instrument included participants’ demographic information, OHISBs, and self-reported health. Data were collected via an online questionnaire using Wenjuanxing software. Table 1 shows the participant demographic characteristics of residence, major, parental education level, and nine OHISBs. Male and female university students were asked if they experienced the following OHISBs while surfing the internet in the past six months. According to their self-reported responses (0 = poor, 1 = good), the frequencies of the nine OHISBs were counted for further analysis.

**Table 1:** Variables definition – Xinyang, Henan, China, 2022.

Variables	Variable definition (0: as references, respectively)
Self-reported health	0 = Poor, 1 = Good
Residence	0 = Rural, 1 = Urban
Major	0 = Science and engineering, 1 = Humanities
Parental education level	0 = Middle school or below, 1 = Doctor/Master/University/College
Finding information about hospitals or doctors	0 = No, 1 = Yes
Finding information about physical exercises	0 = No, 1 = Yes
Finding information about smoking cessation	0 = No, 1 = Yes
Finding health or medical information	0 = No, 1 = Yes
Finding information about drinking cessation	0 = No, 1 = Yes
Reading or sharing health information via social media (e.g., Weibo and WeChat)	0 = No, 1 = Yes
Finding information about nutrition and diet	0 = No, 1 = Yes
Attending a specific disease internet community	0 = No, 1 = Yes
Online reservation of health care projects	0 = No, 1 = Yes

Note: Self-report the following behaviors that you have engaged in while surfing the Internet in the past 6 months. Then, select “0 = No” or “1 = Yes.” Table 1 was referenced from the literature^(18)^ and revised.

Due to binary logistic regression, the variables of major and parental education level were merged, respectively. Specifically, liberal arts and arts were merged into humanities (1 = humanities), and science and engineering were merged into science and engineering (0 = science and engineering). Similarly, parental education level was divided into middle school or below (0 = middle school) and others (1 = doctorate, master’s, university, or college). For each of the nine OHISBs, if the university student experienced an OHISB while surfing the internet in the past six months, this OHI-seeking behavior was defined as “Yes” (1), otherwise it was defined as “No” (0). Items described as 0 are used as references, respectively.

### Data Analysis

SPSS v20 (IBM, Armonk, NY, USA) was used for data analysis, including descriptive statistics (mean, standard deviation [SD], frequency, and percentage) as well as independent sample t-tests and binary logistic regression analysis. Independent sample t-tests were used to examine sex differences in online health search behavior among college students. Additionally, logistic regression models were used to analyze the association between self-reported health and online health information seeking behavior (OHISB) for each gender.

### Ethical Aspects

This study was conducted in accordance with the guidelines of the Helsinki Declaration. All procedures relevant to participants were approved by the Xinyang Normal University Ethics Committee (approval number XFEC-2023-025). Each participant voluntarily agreed to participate after being informed of the study’s objectives and context, and after providing written informed consent regarding privacy and information management policies.

## RESULTS

### Demographic Characteristics

Of the 5,408 university students who participated (see Table 2), 72.2% were female and 27.8% were male. Seventy-seven and a half percent lived in rural areas and 22.5% lived in urban areas, with an average age of 19.75 ± 1.345. Fifty-one point two percent of the students majored in liberal arts, and 28.1% majored in science. Seventy-six point six percent of their parents had a middle school education or less. The main significant characteristics of the participants were female, from rural areas, and majoring in science or engineering, with parents of low educational level.

**Table 2:** Characteristics of the participants (n = 5,408) – Xinyang, Henan, China, 2022.

Characteristics	Frequency	Proportion (%)
Sex		
Male	1,501	27.8
Female	3,907	72.2
Residence		
Urban	1,215	22.5
Rural	4,193	77.5
Age (years)		
Average	19.75 ± 1.345	
Major		
Liberal arts	2,768	51.2
Science	1,519	28.1
Engineering course	561	10.4
Art	560	10.4
Parental education level		
PhD/Masters’ degree	14	0.3
University	763	14.1
College	488	9.0
Middle school or below	4,143	76.6

### Sex Differences Among Ohisbs

There are significant sex differences in online health information seeking behaviors (OHISBs) among Chinese university students, except for finding health or medical information (see Table 3). For both males and females, the top three OHISBs were finding information about physical exercise, reading or sharing health information via social media, and finding information about nutrition and diet, with significant sex differences identified for each. Except for finding health or medical information, all eight other OHISBs had significant sex differences.

**Table 3:** Sex differences among OHISBs (n = 5,408) – Xinyang, Henan, China, 2022.

OHI-seeking behaviors	Male		Female		p	
	Mean	SD	Mean	SD
Finding information about physical exercises	0.52	0.500	0.38	0.486	< 0.001
Reading or sharing health information via social media (e.g., Weibo and WeChat)	0.39	0.485	0.48	0.500	< 0.001
Finding nutrition and diet information	0.38	0.485	0.49	0.500	< 0.001
Finding hospital or doctor information	0.29	0.452	0.24	0.427	< 0.001
Finding health or medical information	0.26	0.436	0.28	0.448	0.114
Finding smoking or drinking cessation information	0.09	0.290	0.02	0.153	< 0.001
Joining a disease-specific online community	0.05	0.227	0.02	0.122	< 0.001
Making online healthcare reservations	0.03	0.159	0.01	0.109	0.002
Finding information about physical exercises	0.03	0.183	0.02	0.134	0.002

Note: A t-test was used.

Thus, this result supports H1.

Overall, females preferred finding information about nutrition and diet, while males preferred finding information about physical exercise.

### Associations Between Self-Reported Health and Ohisbs

Table 4 shows the associations between self-reported health and OHISBs. These associations were obtained through binary logistic regression analysis. The goodness-of-fit test indicated that the binary logistic regression model was appropriate for predicting self-reported health (the Hosmer-Lemeshow test p-range was 0.323–0.644).

**Table 4:** Associations between self-reported health and OHISBs for each sex (OR, n = 5,408) – Xinyang, Henan, China, 2022.

OHI-seeking behaviors	OR (CI 95%)	p
Male		
Finding information about hospitals or doctors	2.171 (1.035–4.551)	0.040
Finding information about physical exercises	0.756 (0.360–1.590)	0.461
Finding information about smoking cessation	0.720 (0.171–3.033)	0.654
Finding health or medical information	1.396 (0.632–3.082)	0.409
Finding information about drinking cessation	1.650 (0.401–6.789)	0.488
Reading or sharing health information via social media (e.g., Weibo and WeChat)	1.941 (0.942–4.002)	0.072
Finding information about nutrition and diet	0.806 (0.382–1.703)	0.573
Attending a specific disease internet community	1.315 (0.207–8.348)	0.772
Online reservation of health care projects	2.396 (0.598–9.607)	0.217
Female		
Finding information about hospitals or doctors	1.785 (1.212–2.628)	0.003
Finding information about physical exercises	0.850 (0.579–1.246)	0.405
Finding information about smoking cessation	0.989 (0.307–3.184)	0.986
Finding health or medical information	1.211 (0.817–1.796)	0.341
Finding information about drinking cessation	1.279 (0.327–5.003)	0.724
Reading or sharing health information via social media (e.g., Weibo and WeChat)	0.722 (0.497–1.051)	0.089
Finding information about nutrition and diet	0.936 (0.649–1.351)	0.724
Attending a specific disease internet community	2.573 (0.877–7.551)	0.085
Online reservation of health care projects	2.491 (1.056–5.876)	0.037

Note: Data are expressed as the odds ratio (OR) and the 95% confidence interval (CI) from a binary logistic regression analysis that includes explanatory and control variables.

OHISBs such as finding information about hospitals or doctors (OR = 2.171, CI95% = 1.035–4.551, p < 0.05) significantly impacted males’ self-reported health. Similarly, OHISBs of finding information about hospitals or doctors (OR = 1.785, CI95% = 1.212–2.628, p < 0.01) and of making online healthcare reservations (OR = 2.491, CI95% = 1.056–5.876, p < 0.05) significantly impacted females’ self-reported health. For both male and female university students, OHISBs of finding information about hospitals or doctors significantly impacted their self-reported health. Thus, this result supports H2.

## DISCUSSION

Our findings suggest that online health care reservations had a significant impact on females’ self-reported health and that finding information about hospitals or doctors had a significant impact on the self-reported health of both females and males. This study fills a gap in the literature on predictive OHISBs for university students’ health. It provides a theoretical basis for targeted intervention measures and could improve the health of university students, especially those with frequent OHISBs, in the future.

An increasing number of health information seekers, including college and university students, are using OHI to answer health-related questions. Among Europeans aged 16 to 74, 55% have sought OHI, which is a 21% increase since 2010. Notably, the rate of OHI seekers in Finland, the Netherlands, Denmark, and Germany exceeds 70%^(19)^. In Greece, 90% of college students have used the internet to seek OHI. Of those students, 85.4% were satisfied with the adequacy of health information found through website searches. 61.2% searched blogs, and 34.2% searched social networking sites^(20)^. College students typically used relevant medical websites and apps to find mental health information or communicate with online doctors^(21)^. In Belgrade, Serbia, 79.6% of adolescents reported that online health information influenced their health decisions to varying degrees, ranging from “a little” to “a lot.”^(22)^ Among Korean undergraduates, OHISBs were positively correlated with their engagement in health-promoting behaviors^(23)^. Overconfidence and self-care practices were identified as significant predictors of whether Italian university students sought health information online, according to Italian research^(24)^.

Sex differences in OHISB have been found among university students, including in this study. In France, young people seeking OHISB are more likely to be female than male^(15)^. Women are more active than men in seeking online information about lifestyle and health^(25)^. When searching for health information online, men care about the comprehensiveness and accuracy of the information^(26)^, while women are more interested in how difficult the information is to read and understand. OHI seekers in France aged 15–30 reported that seeking OHI changed their health behaviors, such as the frequency of medical consultations and how they take care of their health. Additionally, it has been found that OHISB frequency is higher among females and younger individuals, while people with chronic diseases or disabilities are more likely than healthy individuals to perform OHISB searches^(27)^.

There are several limitations to this study that should be acknowledged. First, the study had a cross-sectional design. This design cannot determine causality of the study variables. Second, this study only compared two gender groups, male and female, without including a third gender group. Third gender groups face an extremely complex environment when accessing health services, and their needs and situations are often overlooked in existing research and information. This neglect fails to fully reflect the gender diversity of the real world and may exacerbate misunderstandings and biases toward different gender groups. However, this study was implemented in China. According to current Chinese law, gender registration mainly uses a binary classification system (male or female) at birth, and an official third gender category has not yet been established. Therefore, the study adhered to the local legal framework regarding gender classification. If future legal policies are adjusted to include considerations for the third gender or if research designs include such considerations, they will help reveal health differences between gender groups more comprehensively. Third, this study is limited to self-reported questionnaires from a single university without medical majors, which may introduce biases into the results. Nevertheless, the heterogeneity of the more than 5,000 university students from various grades and majors can provide insights into their OHISBs.

## CONCLUSION

This study reveals that the online health information seeking behavior (OHISB) of Chinese university students affects their self-reported health, with significant sex differences. Actively searching for medical resources is significantly associated with self-reported health, and these findings demand a revision of health promotion strategies. We strongly urge educators, policymakers, and families to adopt sex-differentiated interventions. It is crucial to incorporate practical health information literacy training into the university curriculum, particularly for non-health majors, to empower students to manage their health. Future research should focus on three areas: the causal mechanisms between behavior and health, the role of moderators such as eHealth literacy, and the influence of academic background on these behaviors.

## Data Availability

The entire dataset supporting the results of this study is available upon request to the corresponding author.
