# Supplementary material for: Online health information seeking behaviors and self-reported health among Chinese university students: associations and sex differences
Source: Rev Esc Enferm USP. 2026 Feb 23;60:e20250253. doi: 10.1590/1980-220X-REEUSP-2025-0253en (PMC12928725; doi:10.1590/1980-220X-REEUSP-2025-0253en)
Supplement: Translation

# Translation: Comportamientos de búsqueda de información sanitaria en línea y autoevaluación de la salud entre estudiantes universitarios chinos: asociaciones y diferencias entre sexos

**DOI:** 10.1590/1980-220X-REEUSP-2025-0253es

**Authors:** Jie Chen, Hua Tian

**Affiliations:** 1 Xinyang Normal University, School of Marxism, Xinyang, China.; 2 Xinyang Normal University, College of Tea and Food Science, Xinyang, China

**Keywords:** Interoperabilidad de la Información en Salud, Evaluación en Salud, Características Sexuales, Estudiantes

## Abstract

**Objetivos:**

Explorar las asociaciones y las diferencias entre sexos en lo que respecta a los comportamientos de búsqueda de información sanitaria en línea (CBIOSs) y a la salud autoinformada entre estudiantes universitarios.

**Métodos:**

Estudio transversal y analítico realizado con 5.408 estudiantes universitarios de la Universidad Normal de Xinyang, que respondieron al cuestionario en Wenjunxing como participantes válidos. Se realizaron pruebas t y análisis de regresión logística binaria utilizando SPSS.

**Resultados:**

Se encontraron diferencias entre sexos en los CBIOSs. Las mujeres mostraron mayor preferencia por IOS relacionados con la nutrición alimentaria y la dieta (p < 0,001), mientras que los hombres mostraron mayor preferencia por IOS relacionados con el ejercicio físico (p < 0,001). En cuanto a CBIOSs específicos, buscar información sobre hospitales o médicos (OR = 1,785, [IC95% = 1,212–2,628], p < 0,01) y realizar reservas en línea de proyectos de atención sanitaria (OR = 2,491, [IC95% = 1,056–5,876], p < 0,05) tuvieron impactos significativos en la salud autoevaluada de las mujeres, y buscar información sobre hospitales o médicos (OR = 2,171, [IC95% = 1,035–4,551], p < 0,05) tuvo un impacto significativo en la salud autoevaluada de los hombres.

**Conclusiones:**

Los hallazgos sobre la búsqueda de información sanitaria en línea diferenciada por sexo y los beneficios de la búsqueda proactiva indican que las intervenciones digitales eficaces para estudiantes deben combinar estrategias específicas de género con empoderamiento conductual.

## INTRODUCCIÓN

Desde el inicio de la pandemia de la enfermedad por coronavirus 2019 (COVID-19), cada vez más personas han recurrido a Internet para buscar información relacionada con la salud^(1)^. Internet se ha convertido en una valiosa fuente de información sobre salud, ya que ofrece recursos sobre salud mental^(2)^, salud general^(3)^, relaciones médico-paciente y salud y bienestar. Muchos sitios web y aplicaciones relacionados con la salud proporcionan información sanitaria orientada al consumidor y funciones adicionales^(4)^. La información sanitaria en línea (online health information - OHI) se ha convertido en una de las fuentes de información más importantes^(5)^ para personas de todas las edades que buscan información sobre salud^(6)^. Entre ellas se incluyen foros, blogs, grupos de apoyo en línea y enlaces de recomendación a información sobre todos los aspectos de la salud^(7)^. La información específica sobre salud incluye síntomas, diagnósticos, tratamientos y prevención de enfermedades, detección y evaluación de riesgos. La información general incluye temas como la pérdida de peso, la alimentación saludable y consejos de salud^(8)^. Para los estudiantes universitarios de la Universidad Ondokuz Mayıs que no cursan estudios de ciencias de la salud, las fuentes de información de salud en línea más utilizadas son los foros y los sitios web de hospitales y clínicas privadas, mientras que los sitios web gubernamentales son los menos utilizados^(9)^.

Se considera que los comportamientos de búsqueda de información sobre salud en línea (online health information seeking behaviours - OHISB) tienen un impacto positivo en quienes buscan OHI^(10)^. Según una encuesta realizada en dos universidades del sur de Estados Unidos, el 36,7 % de los estudiantes consideraba que la información sanitaria en línea mejoraba su atención médica de forma significativa o considerable. Un número significativamente mayor de estudiantes mujeres (77,9 %) que de estudiantes hombres (68,6 %) obtenía información sanitaria en línea^(11)^. Un total del 75 % de las personas que buscaban información sobre salud creían que esta tenía un impacto leve o significativo en sus decisiones sobre tratamientos médicos, métodos de mantenimiento de la salud y pensamiento relacionado con la salud^(12)^. En cuanto a la información sobre el nuevo coronavirus, los estudiantes varones eran más propensos que las estudiantes mujeres a creer que Internet por sí solo podía proporcionar toda la información relevante^(13)^. En Nigeria, la presencia de una enfermedad crónica y el género se asociaron significativamente con las OHISB de los estudiantes universitarios no médicos^(14)^. Un estudio mostró asociaciones significativas y diferencias de género entre las OHISB de los estudiantes universitarios chinos y su alfabetización en salud electrónica^(15)^. Sin embargo, los estudios han demostrado que los estudiantes universitarios carecen de los conocimientos y habilidades necesarios para identificar la información incorrecta y los riesgos evidentes de sus comportamientos de búsqueda de información^(4)^. Parte de la información sanitaria en línea (OHI) puede ser inexacta o engañosa, causando dolor y ansiedad innecesarios a los estudiantes universitarios. Por lo tanto, los planes de estudio futuros deberían incluir medidas de intervención adecuadas, como sesiones prácticas, cursos y clases, así como orientación sobre cómo obtener información sanitaria fiable, especialmente para los estudiantes de campos no relacionados con la salud.

Comprender las diferencias de género en los OHISB de los estudiantes universitarios es fundamental para desarrollar intervenciones específicas. La teoría de género contemporánea postula que el género es una construcción social diversa, dinámica y culturalmente sensible, y no simplemente un binario biológico^(14)^. Desde esta perspectiva, excluir las identidades no binarias no refleja la diversidad del mundo real y corre el riesgo de perpetuar los malentendidos y los prejuicios. Sin embargo, este estudio se llevó a cabo en China, donde los marcos legales y sociales son binarios y solo reconocen los géneros biológicos masculino y femenino al nacer, sin un tercer género definido legalmente. Por lo tanto, el alcance de este estudio se limita a un análisis comparativo de hombres y mujeres al nacer y no tiene en cuenta una diversidad de género más amplia^(15)^.

Por lo tanto, se proponen las siguientes hipótesis:

H1: Existen diferencias significativas entre sexos en los OHISB entre los estudiantes universitarios chinos que pueden servir de base para el desarrollo y la aplicación de medidas de intervención específicas^(16)^.

Los estudios han descubierto que los pacientes con mala salud tienden a participar en OHISB con mayor frecuencia y tienen un bajo nivel de alfabetización electrónica en materia de salud; por lo tanto, pueden ser más susceptibles a una OHI poco fiable y a los riesgos para la salud que esta supone. La percepción de malestar podría provocar un mayor nivel de OHISB. Además, los niveles más altos de OHISB están significativamente relacionados con niveles más bajos de salud autoinformados y niveles más altos de angustia mental^(17)^. Sin embargo, pocos estudios han explorado la relación entre los OHISB específicos y la salud de los estudiantes universitarios.

H2: Los OHISB específicos tienen un impacto significativo en la salud autoinformada y están asociados con la salud autoinformada de los estudiantes universitarios.

En resumen, estos hallazgos pueden abordar la falta de investigación sobre las diferencias de género en los OHISB y la relación entre la salud autoinformada y los OHISB específicos^(9)^ entre los estudiantes universitarios chinos. También pueden proporcionar una base teórica para futuras medidas de intervención específicas, especialmente para los estudiantes universitarios de campos no relacionados con la salud.

## MÉTODO

### Diseño del Estudio

Se trata de un estudio transversal no experimental.

### Población, Lugar y Entorno

Este estudio se llevó a cabo entre estudiantes universitarios que cursaban estudios de humanidades, ciencias e ingeniería (no relacionados con las ciencias de la salud) en la Universidad Normal de Xinyang, en la ciudad de Xinyang, provincia de Henan, China. Entre octubre y diciembre de 2022, se reclutó a estudiantes universitarios para que rellenaran de forma voluntaria y libre un cuestionario. Este estudio incluyó 9 cuestionarios OHISB, cuestionarios de salud autoinformada y cuestionarios de información demográfica.

### Criterios de Selección De La Muestra

El estudio reclutó a 5672 estudiantes universitarios, desde estudiantes de primer año hasta estudiantes de último año, para completar un cuestionario anónimo en línea. El cuestionario se creó utilizando el software Wenjunxing. A continuación, se envió el enlace al cuestionario a un grupo de usuarios de WeChat que eran profesores, a quienes se les pidió que lo compartieran con los estudiantes universitarios para reclutar participantes voluntarios. El cuestionario es anónimo y voluntario, y los participantes pueden completarlo libre e independientemente en sus computadoras o teléfonos inteligentes. No hubo recompensa por participar en el estudio. Los participantes eran hablantes nativos de mandarín de entre 18 y 25 años que estudiaban humanidades, ciencias, ingeniería o artes. Se excluyó a quienes no completaron el cuestionario (por ejemplo, quienes dejaron todas las preguntas sin responder, proporcionaron información demográfica incompleta o no eran estudiantes universitarios). Al final, se seleccionaron 5408 estudiantes universitarios (una tasa de respuesta del 95,35 %) como participantes válidos.

### Instrumento de Recolección de Datos

Nuestro instrumento incluía información demográfica de los participantes, OHISB y salud autodeclarada.

Los datos se recolectaron mediante un cuestionario en línea utilizando el software Wenjuanxing. La tabla 1 muestra las características demográficas de los participantes en cuanto a residencia, especialidad, nivel educativo de los padres y nueve OHISB. Se preguntó a los estudiantes universitarios de ambos sexos si habían experimentado los siguientes OHISB mientras navegaban por Internet en los últimos seis meses. Según sus respuestas autoinformadas (0 = malo, 1 = bueno), secontabilizaron las frecuencias de los nueve OHISB para su posterior análisis.

**Tabla 1:** Definición de variables – Xinyang, Henan, China, 2022.

Variables	Definición de variables (0: como referencia, respectivamente)
Salud autodeclarada	0 = Mala, 1 = Buena
Residencia	0 = Rural, 1 = Urbana
Especialidad	0 = Ciencias e ingeniería 1 = Humanidades
Nivel educativo de los padres	0 = Secundaria o inferior 1 = Doctorado/Máster/ Universidad/Escuela superior
Búsqueda de información sobre hospitales o médicos	0 = No, 1 = Sí
Buscar información sobre ejercicios físicos	0 = No, 1 = Sí
Buscar información sobre cómo dejar de fumar	0 = No, 1 = Sí
Buscar información sobre salud o medicina	0 = No, 1 = Sí
Buscar información sobre cómo dejar de beber	0 = No, 1 = Sí
Leer o compartir información sobre salud a través de las redes sociales (por ejemplo, Weibo y WeChat)	0 = No, 1 = Sí
Buscar información sobre nutrición y dieta	0 = No, 1 = Sí
Participar en una comunidad de Internet sobre una enfermedad específica	0 = No, 1 = Sí
Reserva online de proyectos de atención sanitaria	0 = No, 1 = Sí

Nota: Indique los siguientes comportamientos que ha tenido mientras navegaba por Internet en los últimos 6 meses. A continuación, seleccione «0 = No» o «1 = Sí». La tabla 1 se ha tomado como referencia de la bibliografía (18)y se ha revisado.

Debido a la regresión logística binaria, las variables de especialidad y nivel educativo de los padres se fusionaron, respectivamente. En concreto, las artes liberales y las artes se fusionaron en humanidades (1 = humanidades), y las ciencias y la ingeniería se fusionaron en ciencias e ingeniería (0 = ciencias e ingeniería). Del mismo modo, el nivel educativo de los padres se dividió en secundaria o inferior (0 = secundaria) y otros (1 = doctorado, máster, universidad o instituto). Para cada uno de los nueve OHISB, si el estudiante universitario experimentó un OHISB mientras navegaba por Internet en los últimos seis meses, este comportamiento de búsqueda de OHI se definió como «Sí» (1); de lo contrario, se definió como «No» (0). Los elementos descritos como 0 se utilizan como referencias, respectivamente.

### Análisis de Datos

Se utilizó SPSS v20 (IBM, Armonk, Nueva York, EE. UU.) para el análisis de datos, incluyendo estadísticas descriptivas (media, desviación estándar [DE], frecuencia y porcentaje), así como pruebas t independientes y análisis de regresión logística binaria. Se utilizaron pruebas t para muestras independientes para examinar las diferencias de género en el comportamiento de búsqueda de información sobre salud en línea entre los estudiantes universitarios. Además, se utilizaron modelos de regresión logística para analizar la asociación entre la salud autodeclarada y el comportamiento de búsqueda de información sobre salud en línea (OHISB) para cada género.

### Aspectos Éticos

Este estudio se llevó a cabo de acuerdo con las directrices de la Declaración de Helsinki. Todos los procedimientos relevantes para los participantes fueron aprobados por el Comité de Ética de la Universidad Normal de Xinyang (número de aprobación XFEC-2023-025). Cada participante aceptó voluntariamente participar después de ser informado de los objetivos y el contexto del estudio, y después de proporcionar su consentimiento informado por escrito con respecto a las políticas de privacidad y gestión de la información.

## RESULTADOS

### Características Demográficas

De los 5408 estudiantes universitarios que participaron (véase la tabla 2), el 72,2 % eran mujeres y el 27,8 % hombres. El 77,5 % vivía en zonas rurales y el 22,5 % en zonas urbanas, con una edad media de 19,75 ± 1,345. El 51,2 % de los estudiantes se especializaba en humanidades y el 28,1 % en ciencias. El 76,6 % de sus padres tenía estudios de secundaria o menos. Las principales características significativas de los participantes eran ser mujer, proceder de zonas rurales y especializarse en ciencias o ingeniería, con padres de bajo nivel educativo.

**Tabla 2:** Características de los participantes (n = 5408) – Xinyang, Henan, China, 2022.

Características	Frecuencia	Proporción (%)
Sexo		
Masculino	1.501	27,8
Mujer	3.907	72,2
Residencia		
Urbana	1.215	22,5
Rural	4.193	77,5
Edad (años)		
Media	19,75 ± 1,345	
Especialidad		
Humanidades	2.768	51,2
Ciencias	1.519	28,1
Cursos de ingeniería	561	10,4
Arte	560	10,4
Nivel educativo de los padres		
Doctorado/Máster	14	0,3
Universidad	763	14,1
Escuela superior	488	9,0
Secundaria o inferior	4.143	76,6

### Diferencias de Sexo Entre los Ohisb

Existen diferencias significativas entre sexos en los comportamientos de búsqueda de información sanitaria en línea (OHISB) entre los estudiantes universitarios chinos, excepto en lo que respecta a la búsqueda de información sanitaria o médica (véase la tabla 3). Tanto para los hombres como para las mujeres, los tres OHISB principales fueron la búsqueda de información sobre ejercicio físico, la lectura o el intercambio de información sanitaria a través de las redes sociales y la búsqueda de información sobre nutrición y dieta, con diferencias significativas entre sexos en cada uno de ellos. Excepto en la búsqueda de información sanitaria o médica, los otros ocho OHISB presentaban diferencias significativas entre sexos.

**Tabla 3:** Diferencias de género entre los OHISB (n = 5408) – Xinyang, Henan, China, 2022.

Comportamientos de búsqueda de OHI	Hombres		Mujeres		p	
	Media	SD	Media	DE
Búsqueda de información sobre ejercicios físicos	0,52	0,500	0,38	0,486	< 0,001
Leer o compartir información sobre salud a través de las redes sociales (por ejemplo, Weibo y WeChat)	0,39	0,485	0,48	0,500	< 0,001
Búsqueda de información sobre nutrición y dietas	0,38	0,485	0,49	0,500	< 0,001
Búsqueda de información sobre hospitales o médicos	0,29	0,452	0,24	0,427	< 0,001
Búsqueda de información sanitaria o médica	0,26	0,436	0,28	0,448	0,114
Buscar información sobre cómo dejar de fumar o beber	0,09	0,290	0,02	0,153	< 0,001
Unirse a una comunidad en línea específica para una enfermedad	0,05	0,227	0,02	0,122	< 0,001
Realizar reservas sanitarias en línea	0,03	0,159	0,01	0,109	0,002
Buscar información sobre ejercicios físicos	0,03	0,183	0,02	0,134	0,002

Nota: Se utilizó una prueba t.

Por lo tanto, este resultado respalda la hipótesis H1.

En general, las mujeres prefirieron buscar información sobre nutrición y dieta, mientras que los hombres prefirieron buscar información sobre ejercicio físico.

### Asociaciones Entre la Salud Autodeclarada y Los Ohisb

La tabla 4 muestra las asociaciones entre la salud autodeclarada y los OHISB. Estas asociaciones se obtuvieron mediante un análisis de regresión logística binaria. La prueba de bondad de ajuste indicó que el modelo de regresión logística binaria era adecuado para predecir la salud autodeclarada (el rango p de la prueba de Hosmer-Lemeshow fue de 0,323 a 0,644).

**Tabla 4:** Asociaciones entre la salud autoinformada y los OHISB para cada sexo (OR, n = 5408) – Xinyang, Henan, China, 2022.

Comportamientos de búsqueda de OHI	OR (IC 95 %)	p
Hombres		
Buscar información sobre hospitales o médicos	2,171 (1,035–4,551)	0,040
Buscar información sobre ejercicios físicos	0,756 (0,360–1,590)	0,461
Buscar información sobre cómo dejar de fumar	0,720 (0,171–3,033)	0,654
Buscar información sobre salud o medicina	1,396 (0,632–3,082)	0,409
Buscar información sobre cómo dejar de beber	1,650 (0,401–6,789)	0,488
Leer o compartir información sobre salud a través de las redes sociales (por ejemplo, Weibo y WeChat)	1,941 (0,942–4,002)	0,072
Buscar información sobre nutrición y dieta	0,806 (0,382–1,703)	0,573
Participar en una comunidad de Internet sobre una enfermedad específica	1,315 (0,207–8,348)	0,772
Reserva online de proyectos de asistencia sanitaria	2,396 (0,598–9,607)	0,217
Mujer		
Búsqueda de información sobre hospitales o médicos	1,785 (1,212–2,628)	0,003
Buscar información sobre ejercicios físicos	0,850 (0,579–1,246)	0,405
Buscar información sobre cómo dejar de fumar	0,989 (0,307–3,184)	0,986
Buscar información sobre salud o medicina	1,211 (0,817–1,796)	0,341
Buscar información sobre cómo dejar de beber	1,279 (0,327–5,003)	0,724
Leer o compartir información sobre salud a través de las redes sociales (por ejemplo, Weibo y WeChat)	0,722 (0,497–1,051)	0,089
Buscar información sobre nutrición y dieta	0,936 (0,649–1,351)	0,724
Participar en una comunidad de Internet sobre una enfermedad específica	2,573 (0,877–7,551)	0,085
Reserva online de proyectos de atención sanitaria	2,491 (1,056–5,876)	0,037

Nota: Los datos se expresan como odds ratio (OR) y el intervalo de confianza (IC) del 95 % a partir de un análisis de regresión logística binaria que incluye variables explicativas y de control.

Los OHISB, como la búsqueda de información sobre hospitales o médicos (OR = 2,171, IC del 95 % = 1,035-4,551, p < 0,05), tuvieron un impacto significativo en la salud autoinformada de los hombres. Del mismo modo, las OHISB relacionadas con la búsqueda de información sobre hospitales o médicos (OR = 1,785, IC del 95 % = 1,212-2,628, p < 0,01) y con la realización de reservas sanitarias en línea (OR = 2,491, IC del 95 % = 1,056-5,876, p < 0,05) tuvieron un impacto significativo en la salud autodeclarada de las mujeres. Tanto para los estudiantes universitarios como para las estudiantes universitarias, los OHISB relacionados con la búsqueda de información sobre hospitales o médicos tuvieron un impacto significativo en su salud autodeclarada. Por lo tanto, este resultado respalda la hipótesis H2.

## DISCUSIÓN

Nuestros hallazgos sugieren que las reservas de atención médica en línea tuvieron un impacto significativo en la salud autoinformada de las mujeres y que la búsqueda de información sobre hospitales o médicos tuvo un impacto significativo en la salud autoinformada tanto de mujeres como de hombres. Este estudio llena un vacío en la literatura sobre los OHISB predictivos para la salud de los estudiantes universitarios. Proporciona una base teórica para medidas de intervención específicas y podría mejorar la salud de los estudiantes universitarios, especialmente aquellos con OHISB frecuentes, en el futuro.

Cada vez son más las personas que buscan información sobre salud, incluidos los estudiantes universitarios, que utilizan la OHI para responder a preguntas relacionadas con la salud. Entre los europeos de 16 a 74 años, el 55 % ha buscado OHI, lo que supone un aumento del 21 % desde 2010. Cabe destacar que la tasa de personas que buscan OHI en Finlandia, los Países Bajos, Dinamarca y Alemania supera el 70 %^(19)^. En Grecia, el 90 % de los estudiantes universitarios ha utilizado Internet para buscar OHI. De esos estudiantes, el 85,4 % se mostró satisfecho con la adecuación de la información sanitaria encontrada a través de búsquedas en sitios web. El 61,2 % buscó en blogs y el 34,2 % en redes sociales^(20)^. Los estudiantes universitarios solían utilizar sitios web y aplicaciones médicas relevantes para encontrar información sobre salud mental o comunicarse con médicos en línea^(21)^. En Belgrado, Serbia, el 79,6 % de los adolescentes afirmaron que la información sanitaria en línea influía en sus decisiones sobre salud en diversos grados, desde “un poco” hasta “mucho”^(22)^. Entre los estudiantes universitarios coreanos, el OHISB se correlacionaba positivamente con su participación en comportamientos que promovían la salud^(23)^. Según una investigación italiana^(24)^, el exceso de confianza y las prácticas de autocuidado se identificaron como predictores significativos de si los estudiantes universitarios italianos buscaban información sobre salud en línea.

Se han encontrado diferencias de género en el OHISB entre los estudiantes universitarios, incluso en este estudio. En Francia, los jóvenes que buscan OHISB son más propensos a ser mujeres que hombres^(15)^. Las mujeres son más activas que los hombres en la búsqueda de información en línea sobre estilo de vida y salud^(25)^. Al buscar información sobre salud en línea, los hombres se preocupan por la exhaustividad y la precisión de la información^(26)^, mientras que las mujeres están más interesadas en la dificultad de leer y comprender la información. Las personas de entre 15 y 30 años que buscaban información sobre salud en línea en Francia informaron de que dicha búsqueda había cambiado sus comportamientos de salud, como la frecuencia de las consultas médicas y la forma de cuidar su salud. Además, se ha descubierto que la frecuencia de OHISB es mayor entre las mujeres y las personas más jóvenes, mientras que las personas con enfermedades crónicas o discapacidades son más propensas que las personas sanas a realizar búsquedas de OHISB^(27)^.

Hay varias limitaciones en este estudio que deben tenerse en cuenta. En primer lugar, el estudio tenía un diseño transversal. Este diseño no permite determinar la causalidad de las variables del estudio. En segundo lugar, este estudio solo comparó dos grupos de género, masculino y femenino, sin incluir un tercer grupo de género. Los terceros grupos de género se enfrentan a un entorno extremadamente complejo a la hora de acceder a los servicios de salud, y sus necesidades y situaciones suelen pasarse por alto en las investigaciones y la información existentes. Este descuido no refleja plenamente la diversidad de género del mundo real y puede exacerbar los malentendidos y los prejuicios hacia los diferentes grupos de género. Sin embargo, este estudio se llevó a cabo en China. Según la legislación china vigente, el registro de género utiliza principalmente un sistema de clasificación binario (masculino o femenino) al nacer, y aún no se ha establecido una tercera categoría de género oficial. Por lo tanto, el estudio se ajustó al marco legal local en lo que respecta a la clasificación de género. Si en el futuro se ajustan las políticas legales para incluir consideraciones sobre el tercer género o si los diseños de investigación incluyen tales consideraciones, se podrán revelar de forma más exhaustiva las diferencias de salud entre los grupos de género. En tercer lugar, este estudio se limita a cuestionarios autoadministrados de una sola universidad sin carreras de medicina, lo que puede introducir sesgos en los resultados. No obstante, la heterogeneidad de los más de 5000 estudiantes universitarios de diversos grados y especialidades puede proporcionar información sobre sus OHISB.

## CONCLUSIÓN

Este estudio revela que el comportamiento de búsqueda de información sanitaria en línea (OHISB) de los estudiantes universitarios chinos afecta a su salud autodeclarada, con diferencias significativas entre sexos. La búsqueda activa de recursos médicos está significativamente asociada con la salud autodeclarada, y estos hallazgos exigen una revisión de las estrategias de promoción de la salud. Instamos encarecidamente a los educadores, los responsables políticos y las familias a que adopten intervenciones diferenciadas por sexo. Es fundamental incorporar la formación práctica en alfabetización en información sanitaria en el plan de estudios universitario, especialmente para las especialidades no relacionadas con la salud, con el fin de capacitar a los estudiantes para que gestionen su salud. Las investigaciones futuras deberían centrarse en tres áreas: los mecanismos causales entre el comportamiento y la salud, el papel de los moderadores, como la alfabetización en salud electrónica, y la influencia de la formación académica en estos comportamientos.

## Data Availability

El conjunto de datos que respaldan las conclusiones de este estudio está disponible previa solicitud al autor correspondiente.
